# Repurposing clinical drugs is a promising strategy to discover drugs against Zika virus infection

**DOI:** 10.1007/s11684-021-0834-9

**Published:** 2020-12-28

**Authors:** Weibao Song, Hongjuan Zhang, Yu Zhang, Rui Li, Yanxing Han, Yuan Lin, Jiandong Jiang

**Affiliations:** State Key Laboratory of Bioactive Substances and Function of Natural Medicine, Institute of Materia Medica, Chinese Academy of Medical Sciences and Peking Union Medical College, Beijing, 100050 China

**Keywords:** Zika virus, clinical drugs, ZIKV inhibitors, antivirals, repurposing

## Abstract

Zika virus (ZIKV) is an emerging pathogen associated with neurological complications, such as Guillain-Barré syndrome in adults and microcephaly in fetuses and newborns. This mosquito-borne flavivirus causes important social and sanitary problems owing to its rapid dissemination. However, the development of antivirals against ZIKV is lagging. Although various strategies have been used to study anti-ZIKV agents, approved drugs or vaccines for the treatment (or prevention) of ZIKV infections are currently unavailable. Repurposing clinically approved drugs could be an effective approach to quickly respond to an emergency outbreak of ZIKV infections. The well-established safety profiles and optimal dosage of these clinically approved drugs could provide an economical, safe, and efficacious approach to address ZIKV infections. This review focuses on the recent research and development of agents against ZIKV infection by repurposing clinical drugs. Their characteristics, targets, and potential use in anti-ZIKV therapy are presented. This review provides an update and some successful strategies in the search for anti-ZIKV agents are given.

## Introduction

Zika virus (ZIKV) of the genus Flavivirus is responsible for large disease outbreaks attributed to transmission by mosquitoes [1]. ZIKV infection may sometimes lead to severe neurological complications, including microcephaly in fetuses and newborns and a remarkable increase in the number of Guillain–Barré syndrome cases [2,3]. Owing to its volatile epidemics and teratogenic effect, the World Health Organization declared ZIKV as a Public Health Emergency of International Concern in 2016 [4].

Similar to other flaviviruses, ZIKV is a single-stranded positive-sense RNA virus approximately 10 kb in length that encodes a polyprotein [5]. The polyprotein is cleaved into three structural proteins, namely, capsid (C), premembrane (prM), and envelope (E) and seven nonstructural (NS) proteins (NS1, NS2A, NS2B, NS3, NS4A, NS4B, and NS5), which are involved in viral genome replication and virulence [6]. Among these proteins, only NS3 and NS5 have enzymatic activities [7]. NS3 possesses serine protease, RNA helicase, RNA triphosphate, and nucleoside triphosphate enzymatic activities [8]. Along with NS2B, NS3 protease is responsible for the cleavage and posttranslational modification of the virus polyprotein [9]. NS5 consists of two domains: methyltransferase (MTase) at the N-terminal, which is responsible for viral RNA cap methylation; and RNA-dependent RNA polymerase (RdRp) at the C-terminal, which is required for viral RNA synthesis [10]. Furthermore, the crystal structures of NS2B/NS3 and NS5 proteins have been resolved [9–12]. As targeting viral enzymes is a proven antiviral approach, evidenced by clinically used anti-hepatitis C virus (HCV) and anti-human immunodeficiency virus (HIV) drugs, NS2B/NS3 and NS5 are the main drug targets for anti-ZIKV drug discovery [13]. An overview of ZIKV replication in infected cells is presented in Fig. 1, which describes the important drug targets that can be inhibited by the anti-ZIKV agent.

The general strategies used to search for inhibitors against ZIKV currently include viral protein-based screening [14,15], viral replication-based phenotypic screening [16,17], and repurposing of clinically approved drugs [18,19]. Although these strategies have been considered for the identification of anti-ZIKV agents, no specifically

*These authors contributed equally to this work.

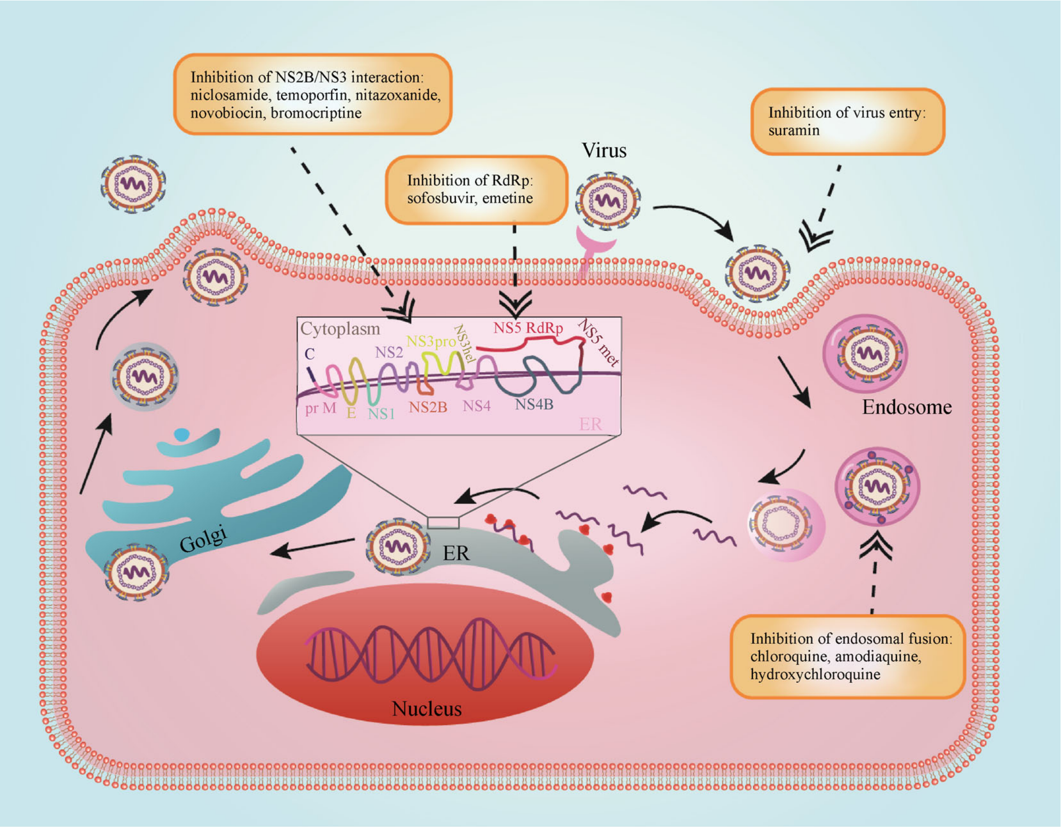

**Fig. 1** Overview of ZIKV replication in the infected cells and the important drug targets that can be inhibited by anti-ZIKV agents discussed in this review.

approved drugs or vaccines are currently available to prevent or treat ZIKV infections. Only a few vaccine candidates and antiviral molecules have progressed to phase I and phase II clinical trials [20,21]. Therefore, repurposing existing drugs is a rapid strategy, which has the advantages of cost-saving and speed in identifying anti-ZIKV agents.

Drug repurposing is defined as "studying drugs that are already approved to treat one disease or condition to assess if they are safe and effective for treating other diseases" [22]. It focuses on US Food and Drug Administration (FDA)-approved drugs, as these drugs have details on potential toxicity, formulation, and pharmacology and drug-like molecules with potential activity [23]. This strategy will address most of the costs and time-consuming hurdles that accompany the drug development process [24]. This approach offers significant achievements to quickly determine ZIKV inhibitors [25,26]. Chloroquine, a well-known antimalarial compound synthesized in 1934, was found to target ZIKV [27,28]. Additionally, sofosbuvir and temoporfin were identified to possess anti-ZIKV activities [29,30]. Researchers often use various methods for drug repurposing, including *in silico*, biological, and experimental approaches. *In silico* drug repurposing merges and analyzes information regarding drug-disease relationships based on various public databases and information from research, reports, and clinical trials [31,32]. Biological approaches have been developed to target multi-factorial complex diseases through systems and network biology [33]. Experimental approaches include the screening of targets, cell assays, animal models, and clinical aspects [34,35]. However, drug repurposing has several disadvantages. Considering the affinity and selectivity for the given primary target, the potency values for novel targets of repurposed drugs are likely to be lower than those observed for the primary target [36]. Conversely, drug repurposing only reduces but does not eliminate the risk of compound development. Therefore, companies need to balance the risks of having a second-in-class drug with lower potency that is not linked to a certain indication.

In this review, we focus on clinically approved drugs that have been evaluated in clinical trials, pre-clinical studies, animal models, or *in vitro* anti-ZIKV tests (Table 1).

## Inhibitors of RdRp

RdRp is the most conserved protein component that plays

**Table 1** Summary of repurposing drugs described in this review

| Name | Structure | Original use | Anti-ZIKV activity | | Mode of action | Drug status | References |
|---|---|---|---|---|---|---|---|
| | | | *In vitro* $EC_{50}$ | *In vivo* | | | |
| Sofosbuvir |  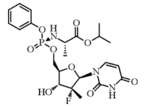 | HCV therapy | 0.41 μmol/L | Active in C57BL/6J mice | Inhibitor of RdRp | Class B FDA-approved drug | [29,34,42] |
| Emetine |  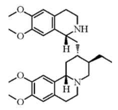 | Intestinal amoebiasis and amoebic liver abscess treatment | 52.9 nmol/L | Active in female SJL mice and Ifnar1$^{-/-}$ mice | Inhibitor of RdRp | FDA-approved drug | [47] |
| Niclosamide |  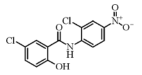 | Worm infections treatment | 0.42–0.54 μmol/L | – | Inhibitor of NS2/NS3 interaction | Class B FDA-approved drug | [19,30] |
| Temoporfin |  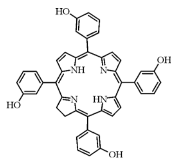 | Squamous cell carcinoma of the head and neck treatment | 0.02–0.027 μmol/L | Active in Balb/C mice | Inhibitor of NS2/NS3 interaction | FDA-approved drug | [30,57] |
| Nitazoxanide |  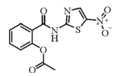 | Various helminthic and protozoal infections treatment | 0.39–1.66 μmol/L | – | Inhibitor of NS2/NS3 interaction | Class B FDA-approved drug | [30,58] |
| Novobiocin |  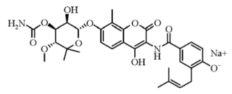 | Antibiotic | 26.12–38.14 μg/mL | Active in dexamethasone-immunosuppressed mice | Inhibitor of NS2/NS3 interaction | Class C FDA-approved drug | [61] |
| Bromocriptine |  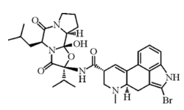 | Galactorrhea and Parkinson's disease treatment | 13 μmol/L | – | Inhibitor of NS2/NS3 interaction | FDA-approved drug | [63] |
| Chloroquine |  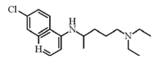 | Malaria and rheumatoid arthritis treatment | 9.82 μmol/L | Active in SJL mice | Disrupting the pH-dependent steps of viral replication | Class C FDA-approved drug | [28,73,74] |

(*Continued*)

| Name | Structure | Original use | Anti-ZIKV activity | | Mode of action | Drug status | References |
|---|---|---|---|---|---|---|---|
| | | | *In vitro* $EC_{50}$ | *In vivo* | | | |
| Hydroxy-chloroquine |  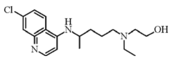 | Antimalarial | – | Active in WT pregnant mice | Alkalizing intracellular acidic organelles; reducing autophagic activity | Class C FDA-approved drug | [84,85] |
| Mefloquine |  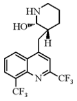 | Antimalarial | 3.6–10 μmol/L | – | – | Class B FDA-approved drug | [18,92] |
| Amodia-quine |  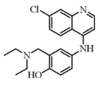 | Antimalarial | 2.8 μmol/L | SCID-beige mice | Targeting an early step of viral replication | Class C FDA-approved drug | [75,94] |
| Azithromy-cin |  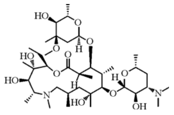 | Antibiotic | 2–15 μmol/L | – | – | FDA-approved drug | [98,100] |
| Daptomycin |  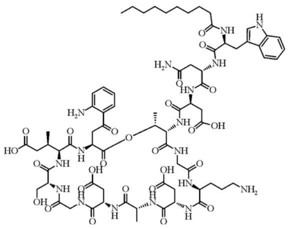 | Antibiotic | 1.0 μmol/L | – | – | Class B FDA-approved drug | [18,102] |
| Emricasan |  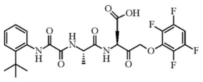 | Hepatic injury and liver fibrosis treatment | – | – | Inhibiting caspases | Phase II clinical trials | [19] |
| Suramin |  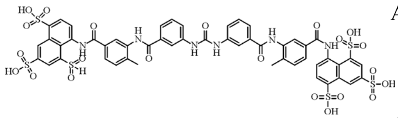 | African trypanos-omiasis and adult *Onchocerca* treatment | 39.8 μmol/L | – | Affecting virus attaching stage and the release of infectious progeny | Investigational | [111] |

an essential role in viral replication. It is considered a promising target for anti-ZIKV drugs.

Sofosbuvir is a successful drug targeting RdRp that is used for HCV therapy [37,38]. It is a uridine nucleotide prodrug that is triphosphorylated intracellularly and can be metabolized to the active 5-triphosphate form, 2-deoxy-2-α-fluoro-2-β-C-methyluridine-5-monophosphate (PSI-7409), in the liver [39,40]. Sofosbuvir is a potent inhibitor of HCV RdRp [41]. Considering its low side effects, good oral administration, and tolerance due to its high potency, sofosbuvir has attracted an increasing amount of attention for development as a direct-acting antiviral drug [38]. Sofosbuvir reportedly inhibited ZIKV replication in Huh-7 cells, with an $EC_{50}$ (half-maximal effective concentration) of 0.41 μmol/L [12,29,34,42]. However, sofosbuvir showed no inhibitory effect on ZIKV replication in Vero and A549 cells. This finding might be correlated with different intracellular concentrations of the active triphosphate metabolite of sofosbuvir, which was 11–342 times higher in Huh-7 cells than in Vero and A549 cells [42]. Sacramento *et al*. reported that sofosbuvir triphosphate inhibited ZIKV RdRp activity, with a half maximal inhibitory concentration ($IC_{50}$) of 0.38–7.3 μmol/L [29,43]. Furthermore, sofosbuvir might increase A-to-G mutations in the viral genome, which is due to anti-ZIKV activity [29]. However, the mutation at S604T of ZIKV RdRp could confer resistance to sofosbuvir [43]. Sofosbuvir protected mice against ZIKV infection and increased survival rates [34]. Sofosbuvir is a class B FDA-approved drug, which implies that it presents no risk to animal fetus. Sofosbuvir's antiviral activity in human neural progenitors and brain organoids demonstrated that it might be a promising drug for clinical ZIKV therapy [12,29].

Emetine, an antiprotozoal agent, is used for intestinal amebiasis and amoebic liver abscess treatment [44–46]. Yang *et al*. observed that emetine possessed anti-ZIKV activity in HEK293 cells, with an $EC_{50}$ of 52.9 nmol/L *in vitro*, and reduced levels of ZIKV in both female SJL mice and Ifnar1$^{-/-}$ mice [47]. Emetine is a non-nucleoside compound that directly inhibited ZIKV NS5 RdRp activity, with an $IC_{50}$ of 121 nmol/L. Moreover, emetine inhibited viral entry by inhibiting lysosome activity [47]. However, emetine has demonstrated potential toxicity toward the fetus, indicating that its use should be avoided during pregnancy [35]. In addition, 10-undecenoic acid zinc salt (UA) was another non-nucleoside drug that inhibits ZIKV replication by targeting RdRp [48]. UA is commonly used in clinics to treat fungal infections [49]. The anti-ZIKV activity of emetine and UA is currently at the *in vitro* stage. The safety of emetine and UA for pregnancy and newborns needs further evaluation.

Some nucleoside analogs have presented good inhibitory effects on ZIKV by targeting RdRp, including BCX4430, NITD008, and 7-deaza-2′-C-methyladenosine (7DMA). BCX4430 inhibited ZIKV replication with an $EC_{50}$ of 3.8 – 11.7 μg/mL *in vitro* and showed protective effects on the AG129 mouse model with ZIKV infection [50]. BCX4430 is currently in phase I clinical trial to evaluate its safety, tolerability, and pharmacokinetics [25,50]. NITD008 and 7DMA exhibited antiviral activity against ZIKV *in vitro* in the micromolar and submicromolar ranges [25]. However, both 7DMA and NITD008 failed during clinical trials [51].

## NS2B/NS3 protease inhibitor

The protease complex, NS2B/NS3, plays an important role in the hydrolysis of ZIKV polyproteins into functional formats. The cleaved proteins, thus function in the process of viral propagation and maturation. In the NS2B/NS3 complex, the partial residues (residues 49–95) in NS2B could help NS3 cleave vital polyproteins more effectively [52–54]. The unlinked construct of NS2B/NS3 is a promising tool for drug discovery [55]. Thus, inhibitors that block the NS2B/NS3 interaction could be promising anti-ZIKV drugs.

Niclosamide, an orally bioavailable salicylanilide approved by the FDA, has been used to treat worm infections [19]. It was reportedly a broad-spectrum flavivirus inhibitor. As a category B drug, niclosamide has an $LD_{50}$ (median lethal dose) of 5 g/kg in rats. A previous study indicated that niclosamide inhibited the NS2B/NS3 interaction in various flaviviruses, presenting an $IC_{50}$ of 12.3 ± 0.6 μmol/L, while the $EC_{50}$ against various flaviviruses ranged between 0.4 and 1.1 μmol/L [56]. However, the $CC_{50}$ (50% cytotoxic concentration) of niclosamide was 4.8 μmol/L, suggesting an unsatisfactory therapeutic index. Researchers observed that the $EC_{50}$ of niclosamide was 0.22 μmol/L for ZIKV production in human astrocytes [19]. Importantly, niclosamide reduced viral loads in both infected men and non-pregnant women and could protect humans against ZIKV-related complications, such as Guillain–Barré syndrome.

Temoporfin, a photosensitizer drug, has been approved for the treatment of head and neck squamous cell carcinoma [57]. A drug repurposing investigation revealed that temoporfin inhibits ZIKV post-infection. The inhibitory efficiency of temoporfin was almost identical at different ZIKV post-infection times [56]. Furthermore, temoporfin interfered with ZIKV production at approximately 40 nmol/L in ZIKV-infected human placental epithelial cells (HPECs). Protein thermal shift assays (PTSA) and surface plasmon resonance (SPR) confirmed the inhibition of NS2B/NS3 interaction. In animal models, temoporfin-treated Balb/C mice showed a 100-fold reduction in ZIKV-induced viremia. In the lethal A129 mouse model, temoporfin-treated animals survived without any neurological damage after ZIKV infection.

Nitazoxanide, an antiparasitic drug used to treat various helminthic and protozoal infections, possesses broad-spectrum antiviral activity against viruses, including flaviviruses [58]. A previous report revealed that nitazoxanide effectively inhibited ZIKV infection in HPECs by reducing protein expression and viral RNA replication [56]. Studies were performed on human neuronal progenitor cells (hNPCs) and the human-induced pluripotent stem cell (iPSC) line HDF9 to investigate nitazoxanide's protective effect. Nitazoxanide markedly reduced ZIKV titers in hNPCs and HDF9 iPSCs cells. Further studies showed that nitazoxanide bound to the NS3 protease domain with an affinity of 7.3 μmol/L, indicating its potent ability to inhibit the NS2B/NS3 interaction. Although studies involving pregnant women remained elusive, nitazoxanide reportedly did not affect human fertility or harm the fetus in rats and rabbits [59].

Novobiocin, an antibiotic derived from *Streptomyces*, is an FDA-approved pregnancy category C drug [60]. As an aminocoumarin antibiotic, novobiocin targets the GyrB subunit of bacterial DNA gyrase to exert its antibacterial effects [61,62]. Novobiocin disrupted NS2B/NS3 interaction and had an $IC_{50}$ of 14.2 μg/mL. The viral titer assay showed that the $EC_{50}$ in Vero and Huh-7 cells was 26.12 μg/mL and 38.14 μg/mL, respectively. A plaque reduction assay revealed that novobiocin achieved 100% plaque reduction at 50 μg/mL. Molecular docking between novobiocin and the NS2B/NS3 protein showed that three hydrogen bonds were formed at NS2B/NS3 interaction sites, namely the MET51 (NS2B residue), SER81 (NS2B residue), and LYS54 (NS3 residue). In novobiocin-treated mice models, ZIKV viral loads in the blood and most major organ tissues were dramatically reduced than in untreated models.

Bromocriptine is a potent dopamine D2/D3 receptor agonist used to treat galactorrhea and Parkinson's disease [63,64]. As an FDA-approved pregnancy category B drug, it is considered safe in pregnant women [28,34]. Bromocriptine had an $EC_{50}$ of 13 μmol/L in ZIKV-infected Vero cells and was used as an agent to treat ZIKV infections in combination with Intron A (interferon-α2b). The data showed that the combination of bromocriptine and Intron A exhibited a synergistic effect on ZIKV infection. Other studies revealed that bromocriptine interfered with ZIKV replication through a post-entry mechanism. Molecular modeling and a fluorescence-based protease inhibition assay showed that bromocriptine disrupted the ZIKV NS2B/NS3 interaction in a non-competitive manner, presenting an $IC_{50}$ of 21.6 μmol/L.

Although small-molecule inhibitors targeting the active site NS2B/NS3 are available, further chemical modifications might be required to improve the potency of these inhibitors [65].

## Antimalarial (quinoline derivatives)

An increasing number of studies observed that some antimalarial drugs possess *in vitro* and *in vivo* anti-ZIKV activities, including mefloquine, chloroquine, amodiaquine, and hydroxychloroquine.

Chloroquine (CQ), a 4-aminoquinoline derivative, is an inhibitor of autophagy and Toll-like receptors (TLRs) and is widely used to treat malaria and rheumatoid arthritis [66–68]. It can also inhibit various viral infections, such as HIV, dengue virus, Japanese encephalitis virus, and influenza virus, by disrupting the pH-dependent steps of viral replication [69–72]. CQ suppressed *in vitro* ZIKV replication in Vero cells with an $EC_{50}$ of 9.82 μmol/L [28,73–75]. It reversed morphological changes induced by ZIKV infection in mouse neurospheres [28]. CQ interfered with the early stage of the ZIKV replication cycle in the fusion of envelope proteins with the endosome membrane. Li *et al.* found that CQ inhibited ZIKV infection *in vitro* by blocking virus internalization [73]. In an animal model, CQ protected fetal mice from microcephaly caused by ZIKV infection. Using interferon signaling-competent SJL mice, Shiryaev *et al.* demonstrated that CQ attenuated vertical transmission, which reduced the ZIKV load in the fetal brain by over 95% [74]. CQ is a class C FDA-approved drug that can cross the placental barrier [76]. Enhanced permeability of the placental barrier benefits therapy in pregnant women.

Hydroxychloroquine (HCQ), a hydroxyl analog of chloroquine, is a class C FDA-approved drug that is used to treat malaria, systemic lupus erythematosus, and rheumatoid arthritis [68,77–79]. Both HCQ and CQ are derivatives of a 4-aminoquinoline nucleus [80]. HCQ is proposed to be a safer CQ alternative [81]. The concentrations of HCQ in the brain are 4–30 times higher than in plasma, suggesting a favorable pharmacokinetic profile against ZIKV infection in hNPCs [74,82]. By alkalizing intracellular acidic organelles, HCQ exerts antibacterial and antiviral activities [83,84]. Furthermore, HCQ blocks the viral entry step and protein glycosylation [84]. Cao *et al.* observed that HCQ could reduce ZIKV infection in the mouse placenta and relieve placental damage in the fetal head [85]. This result might be associated with decreased placental autophagy, which limits vertical maternal-fetal transmission. Kumar *et al.* indicated that HCQ suppressed ZIKV replication by reducing the NS2B/NS3 protease activity [52]. A normal HCQ dosage during pregnancy is not related to fetal malformations [86] and not associated with other adverse pregnancy outcomes, such as stillbirth, low birth weight, and prematurity. However, the current results are insufficient to evaluate its fetotoxicity.

Mefloquine (MQ), a quinine derivative, is a class B FDA-approved drug widely used in malaria prevention [87,88]. Additionally, MQ has anti-cancer, antituberculosis, and antiviral activities [89–91]. Barbosa-Lima *et al*. identified that MQ inhibited ZIKV replication in Vero and HeLa cells, with $EC_{50}$ values of 3.6 μmol/L and 10 μmol/L, respectively [18,92]. However, MQ was found to be cytotoxic in an hNSC cell line (K048) [18]. MQ demonstrated better blood-brain barrier (BBB) penetration than CQ [66]. The anti-ZIKV activity of MQ requires further evaluation.

Amodiaquine (AQ), a 4-aminoquinoline derivative, is another inhibitor of autophagy for malaria therapy [93]. AQ reportedly inhibited ZIKV replication in hNPCs cells, with an $EC_{50}$ of 2.8 μmol/L *in vitro,* and inhibited ZIKV infection in the SCID-beige mouse brain *in vivo* [94]. A report by Han *et al*. revealed that AQ targeted an early step of viral replication [75]. Importantly, AQ is safe during pregnancy and has been used to inhibit the Ebola virus at clinically relevant doses [95]. However, AQ has been restricted in many fields due to its hepatic and hematological toxicities [96]. Therefore, the application of AQ for ZIKV infections requires further investigation.

## Antibiotics

Azithromycin (AZ) is a macrolide antibiotic with no side effects on pregnancy and fetal development [97]. By screening FDA-approved compounds for anti-ZIKV treatment in a glial cell line, Retallack *et al*. found that AZ inhibited viral production and virus-mediated cell death [98]. In U87 cells, AZ reduced ZIKV infection, presenting an $EC_{50}$ of 2–3 μmol/L, whereas in human pluripotent stem cell (hPSC)-derived astrocytes, the $EC_{50}$ was 15 μmol/L. AZ was found to reach 19–151 ng/mL in fetal tissues and the adult human brain [99,100]. The mechanism underlying the action of AZ against ZIKV remains unclear.

Daptomycin is a cyclic lipopeptide antibiotic with potent antibacterial activity against skin and bloodstream infections [101–103]. Daptomycin inhibits ZIKV infection with an $EC_{50}$ of 1.0 μmol/L in Huh-7 cells, but the inhibitory activities in HeLa and JEG3 cells were weak [18]. Furthermore, daptomycin reduced ZIKV replication in hNSC and human amnion epithelial cells (hAECs). The concentration of daptomycin in plasma ranged between 19 and 199 μg/mL [104]. Although daptomycin is a class B FDA-approved drug, limited case reports in neonates have been recorded [102]. No reports regarding the anti-ZIKV activity of daptomycin *in vivo* are currently available.

## Others

By screening drugs for repurposing, several compounds that could inhibit ZIKV infection have been identified, and the various mechanisms are presented.

Emricasan, a selective pan-caspase inhibitor, has been used to treat ZIKV infection in combination with other anti-ZIKV-replication drugs [105,106]. Emricasan inhibited neural cell death induced by caspases but failed to inhibit ZIKV replication [107]. Subsequent studies observed that PHA-690509, a cyclin-dependent kinase inhibitor, possessed good inhibitory activity on ZIKV proliferation. Combination of emricasan and PHA-690509 showed synergistic effects on ZIKV infection. Emricasan could induce the recovery of ZIKV-infected cells by inhibiting caspase when combined with PHA-690509. Emricasan is currently undergoing phase II clinical trials [108,109].

Suramin is not approved by the FDA; it is a polyanionic compound used to treat African trypanosomiasis and kill the adult *Onchocerca* via an unknown mechanism [110]. Suramin could protect Vero cells against ZIKV-induced death, presenting an $EC_{50}$ of 39.8 μmol/L. Albulescu *et al.* demonstrated that suramin could decrease the intracellular ZIKV RNA copies by interfering with both the virus attachment and release stage [111].

After screening 774 FDA-approved drugs, mycophenolic acid, sertraline, and mefloquine were found to be effective in inhibiting ZIKV infection in Huh-7 cells [26]. HeLa and JEG3 cells were also used for further anti-ZIKV investigations. Mycophenolic acid, an immunosuppressant drug, could inhibit ZIKV infection in HeLa and JEG3 cells. In hAECs, both sertraline and mefloquine exhibited strong inhibition against ZIKV infection at 16 μmol/L; none of these drugs were cytotoxic at this concentration [18].

## Conclusions

Identifying novel therapeutic activities of existing drugs is a rapid approach to curb emergency outbreaks of ZIKV infections. Several clinically approved drugs manifest anti-ZIKV activities *in vivo* and are now undergoing clinical trials [74,85]. We discussed the features of these drugs for their potential use in ZIKV infections. Considering that the unique patients include pregnant women and newborns, further work is needed to complete the clinical trials of these drugs. The risk of repurposing drugs needs to be weighed against the risk of no treatment. Moreover, some clinically approved drugs showed anti-ZIKV activities at impracticable inhibition concentrations ($>$ 100 μmol/L) or possessed cytotoxicity, indicating that they could never be utilized in pregnant women and newborns [35,61]. Thus, the immediate use of these repurposed clinical drugs in humans seems unlikely. Medicinal chemistry approaches should be used to improve or optimize these drugs. Additionally, further development of novel anti-ZIKV compounds and combination therapies is needed to treat ZIKV infections.

By repurposing clinical drugs, we are advancing the fight against ZIKV. We believe that the development of highly effective anti-ZIKV drugs is possible.

## Acknowledgements

This work was supported by CAMS Major Collaborative Innovation Project (No. 2016-I2M-1-011), National Natural Science Foundation of China (No. 81773784), Beijing Nova Program (No. Z181100006218075), Basic Scientific Research Program of CAMS (No. 2018RC350005), and Drug Innovation Major Project (No. 2018ZX09711001-002-002).

## Compliance with ethics guidelines

Weibao Song, Hongjuan Zhang, Yu Zhang, Rui Li, Yanxing Han, Yuan Lin, and Jiandong Jiang declare that they have no financial conflicts of interest. This manuscript is a review article and does not involve a research protocol requiring approval by the relevant institutional review board or ethics committee.

**Open Access** This article is licensed under a Creative Commons Attribution 4.0 International License, which permits use, sharing, adaptation, distribution and reproduction in any medium or format, as long as you give appropriate credit to the original author(s) and the source, provide a link to the Creative Commons license, and indicate if changes were made.

The images or other third party material in this article are included in the article's Creative Commons license, unless indicated otherwise in a credit line to the material. If material is not included in the article's Creative Commons license and your intended use is not permitted by statutory regulation or exceeds the permitted use, you will need to obtain permission directly from the copyright holder.

To view a copy of this license, visit https://creativecommons.org/licenses/by/4.0/.
